# The Challenge of “Defining” Emotions

**DOI:** 10.3390/brainsci15121261

**Published:** 2025-11-25

**Authors:** Andrew Ortony

**Affiliations:** Department of Psychology, Northwestern University, Evanston, IL 60208, USA; ortony@northwestern.edu

**Keywords:** affect, appraisals, cognitions, necessary characteristics, valence, emotion intensity, bodily feelings, facial expressions

## Abstract

Taking a cognitive perspective on emotions as generally exemplified by appraisal theories, I suggest that attempts to “define” emotions is a theoretical exercise whose goal should be to specify necessary and jointly sufficient conditions for something to be an emotion. To this end, I advance arguments in support of the proposal that genuine emotions have the five necessary characteristics of being (i) intentional (i.e., about something), (ii) personally significant, (iii) valenced, (iv) consciously experienced, and (v) insuppressible. Collectively, these properties distinguish emotions from other kinds of mental states. I also argue that attempts to define emotions should resist the temptation to incorporate into definitions characteristics of emotions that are not always present, even though, when they are present, those characteristics may be typical and highly salient. It is suggested that two characteristics that are routinely taken to be constitutive of emotions—bodily changes and facial expression—are just such characteristics; they are typical and salient but not in fact necessary as evidenced by the fact that many (especially low intensity) emotions occur without them.

## 1. Introduction

That emotions are ill-defined and poorly understood has been acknowledged for decades, especially by psychologically-oriented emotion theorists (e.g., [1,2,3,4,5,6,7,8]), a fact that was the impetus behind this special edition of *Brain Sciences*. But what would it mean to “define” emotions? It certainly would not mean to define the word “emotion” as used in everyday discourse. That is the task of lexicographers, not of emotion theorists. Indeed, it is largely because the everyday use of the word “emotion” is too vague for scientific purposes that emotion theorists seem not to agree about what emotions are. In fact, lack of clarity about the referents of psychological constructs is quite a general problem in psychology [9]. Addressing important questions about what emotions do and how they do it presupposes that we agree about what emotions are, which we don’t. What is needed is an acceptable characterization of emotions which, while reasonably compatible with the everyday understanding of the emotion concept, is precise, scientifically sound, and clinically useful. The task of providing such a characterization is an exercise in theoretical psychology rather than an empirical enterprise. In what follows, I shall address this task by undertaking a conceptual analysis of the emotion construct. Primarily on the basis of a priori reasoning, I shall propose what I consider to be plausible necessary and jointly sufficient conditions for something to count as an emotion, thereby offering, for future use by affect scientists, a consistent criterion for determining whether something is or is not an emotion.

Interestingly, professional societies often take on the task of specifying precisely what it is that key terms in their domains refer to. For example, the everyday use of the word “planet” includes the idea that a planet is a large body that revolves around the sun or around some other star. While surely a reasonable account of the everyday use of the word, for astronomers it was problematic in that it allowed various kinds of objects to implausibly count as planets. Accordingly, in 2006 the International Astronomical Union (IAU) defined the term for scientific purposes specifying three necessary conditions for an object to count as a planet, conditions which famously led to the demotion of Pluto from planet to “dwarf” planet. A second example pertains to the nature of pain, a common English dictionary definition of which is something like “a distressing sensation in a particular part of the body” but this was too vague for scientific and medical purposes. Although the International Association for the Study of Pain (IASP) already had a formal definition that it had approved in 1979, even that was found lacking, so 40 years later it approved (and justified) a modified definition of pain as “an unpleasant sensory and emotional experience associated with, or resembling that associated with, actual or potential tissue damage” [10]. Perhaps the International Society for Research on Emotion, should follow the examples of the IAU and IASP.

It is important to understand that attempting to specify characteristics that are necessary and sufficient for something to be an emotion means offering reasons for why it makes sense to stipulate that anything that is an emotion should have all of those characteristics. It is a proposal for how to constrain and make precise the emotion concept. This means, to take one of the characteristics that I shall propose as an example, that when I suggest that for something to be an emotion it must be intrinsically valenced, I am not saying that being intrinsically valenced is a fact about emotions, but rather that a formal characterization of the emotion construct should acknowledge valence as an inclusion criterion for the category of emotions. Furthermore, to the extent that it is not possible to specify sufficiency requirements for something to plausibly count as an emotion, it should, at a minimum, be possible to specify necessary characteristics which allow near-miss non-exemplars to be distinguished from legitimate exemplars (as astronomers were able to distinguish Pluto from Earth, Mars, Saturn, etc.).

I approach the task of specifying plausible candidates for necessary characteristics for something to count as an emotion from a cognitive science perspective. Specifically, my orientation is that of an account of emotion sometimes referred to as the OCC model, so-called after its three authors, Ortony, Clore and Collins [11], a model which is an instance of a class of widely held emotion theories known as (cognitive) appraisal theories (e.g., [8,12,13,14,15,16,17]). To set the stage, a brief digression into the basic ideas behind appraisal theories will be helpful. The central tenet of such theories is that as we go about our daily lives we are always evaluating and forming mental representations of the things that we perceive as going on in and around us in terms of their relevance to our past, present, and future concerns [18]. These appraisals of our worlds—these cognitive construals—underlie our emotions, with different emotion types being grounded in different kinds of appraisals. The same situation often can be construed in more than one way (even by the same individual). So, for example, construing a situation as (something like) the successful avoidance of a prospective harm, is a condition for the emergence of one type of emotion (which in English we might call “relief”) whereas the same situation construed as the confirmation of a prediction could underlie the emergence of a different emotion type (e.g., gratification). The plausibility of this general approach is particularly obvious when one considers the different emotions experienced by the participants in competitive sporting events. Take, for instance, the 2022 World Cup final in Qatar between Argentina and France (a game which, incidentally, had a world-wide viewing audience of close to 1.5 billion people). With the outcome still undecided after 120 min of grueling football, Argentina finally defeated France in a very suspenseful penalty shootout. Because the players of the opposing teams were focusing on [and therefore evaluating (i.e., appraising)] the outcome in terms of their different wants and want-nots, likes and dislikes, and so on, their emotions in response to the same event were very, very different. At a minimum, the Argentinians likely appraised the event of their winning the match as something along the lines of the attainment of a highly desirable outcome, while their defeated French opponents construed it as the failure to attain the same. The different ways of appraising—the different construals of—the same event were the foundation of the different emotions (e.g., joy versus disappointment) experienced by the players (and to a great extent also the supporters) of the two teams. They felt the way they did because they construed the event the way they did.

Before turning to the main concern of this article—the postulation of necessary conditions for something to be an emotion—I need to raise three important points. First, I take emotions to be similar to thoughts, perceptions, intentions, and wants, in that whatever their differences, they are all members of the category of mental states. Organisms without minds (and brains) might have reflexes or at least exhibit reflex-like behaviors, but they cannot have (i.e., experience) what we would consider to be emotions. Second, it is helpful to make a clear distinction between emotions and *affect*. I take affect to be a general construct pertaining to evaluation, so while all emotions are affective states, not all affective states are emotions. For example, the state that my colleagues and I have characterized as *undifferentiated affect* is not an emotion [11]. It is just a general, nonspecific, positive or negative state, often inaccessible to consciousness (see also [19] and Russell [20] on *Core Affect*). In viewing, affect as a superordinate construct pertaining to evaluation, I am in general agreement with the position of Peter Walla and colleagues in their article in this special issue of this journal [21].

Finally, in any discussion of emotions, it is important to distinguish between particular instances of emotions and what I call *emotion types*. To understand the difference, notice that although the English language has many words that refer in one way or another to anger (e.g., “aggravated”, “annoyed”, “enraged”, “irritated”, “livid”, “peeved”), there is only one word that refers to relief. So, while we might refer to a particular instance of anger using one of the many anger words, they all refer to (different aspects or intensities of) the one general emotion type, whereas “relief” is the only word available in English to refer to the myriad subtly different kinds of instances of the emotion type that in OCC we characterize as (a positive feeling about) the disconfirmation of an envisaged undesirable event [11] (p. 103). A key implication of the lack of a reliable mapping from emotion types to natural language terms is that emotion types need to be characterized in terms of different kinds of underlying cognitions rather than in terms of the emotion words found in any particular language.

## 2. Five Essential Characteristics of Emotions

### 2.1. Intentionality

As already indicated, along with most psychologically-oriented emotion theorists, I take the view that evaluative appraisals are the cognitive underpinnings of emotions. Consider, for example, a young man who is proud of having saved a child from drowning. The man construes the situation in terms of his belief that he saved a child from drowning, which he evaluates as a good, probably praiseworthy, act. The point to be emphasized here is that the man’s appraisal of what he did is a cognition (in this case, a conscious belief) that has specific *content*, and in general, regardless of their fidelity to the facts and regardless of whether or not we are aware of them, such cognitions always have content, or objects—they are *about* something. The objects of appraisals are representable linguistically as the complements of cognitive verbs such as “anticipate”, “believe”, “contemplate”, “envision”, “imagine”, and so on, verbs that express propositional attitudes [22]. The technical (philosophical) term for this feature of cognitions—having objects, or being about something—is *intentionality*, and because the cognitions that subserve emotions are intentional, emotions themselves are intentional. If one is relieved, or sad, or angry, one is relieved that something specific did (or didn’t) happen, sad about something specific, or angry about something (or with someone, etc.). On this view, a person who reports being sad about things in general (i.e., about nothing in particular) is better considered to be reporting a mood rather than a specific emotion. Furthermore, although a detailed justification is not possible here, I believe that a case can be made for the general statement that the object of an emotion is always the same as the object of the construal that is its cognitive basis. So, if the man’s belief about his aquatic heroics is the basis of his pride, the object of that emotion will be the same as the object of the underlying cognition, namely, saving the child from drowning.

Importantly, not every construal results in an emotion. Although the existence of an emotion necessitates the existence of a corresponding cognition, the converse is not true. The existence of a cognition does not guarantee that an emotion will emerge (hence the caveat towards the end of the previous paragraph indicting the possible conditionality of the man experiencing pride). As will be discussed in the next section, for an emotion to arise other necessary conditions have to be satisfied, including the fact that whatever it is that the emotion is about has to somehow matter to the person experiencing the emotion.

One advantage of taking intentionality to be a necessary characteristic of emotions is that it helps us to distinguish emotions from moods (e.g., irritable, joyful). Even though one may know the cause of a mood, moods are not *about* their causes, and in cases where moods appear to be about something (e.g., as in clinical depression), they aren’t about anything in particular. Thus, whereas you might be depressed (or sad, or dejected) about the fact that the government failed to pass a piece of legislation you really hoped would pass (emotion), it seems odd to say that your depressed mood is about the failed legislation, even though the failure to pass the legislation may well have been a contributing cause of your depressed mood (because you view things in general as being a mess). Interestingly, most emotion types do not allow corresponding moods. We do not think that a person can be in a proud mood, or a relieved mood, or a resentment mood. In view of the above, it seems reasonable to restrict the emotion construct to mental states whose objects are distinct and specifiable, and to treat cases in which there is no distinct and specifiable object, or no object at all, as moods. Moods are perhaps best viewed as diffuse affective states which give affective coloration to most of what is being experienced, while also lowering the threshold for getting into mood-compatible emotional states.

The idea that moods are not necessarily intentional is a more principled way of distinguishing moods from emotions than, for example, duration which, although frequently cited as the key difference between moods and emotions, is not a reliable distinguishing characteristic because not all moods last longer than all emotions (some moods pass in a few minutes, and some emotions can last for hours). Intentionality also provides a ready way of distinguishing emotions from (emotional) traits (e.g., aggressive, greedy), which, in spite of their emotional associations, are not emotions. Just as being in a bad mood does not necessitate that one is irritable about anything in particular, so too does being aggressive not necessitate that one is aggressive towards anyone in particular. Even more obviously, traits are not states. Traits are merely tendencies, proclivities, summaries of behaviors; they pertain to particular domains, to be sure, but they aren’t about anything specific.

### 2.2. Personal Significance

I take it as self-evident that for an emotion to arise, one has to *care* about whatever it is that the emotion is about, meaning that it makes no sense to say that one has an emotion about something about which one harbors an attitude of indifference [23,24]. This is what I mean by saying that for a mental state to count as an emotion its object must have (some degree of) *personal significance* for the experiencing individual. Thus, I am taking personal significance to be a measure of something like subjective importance—the degree to which someone cares about something. Consider as a simple illustration, the case of a person of modest means who comes to realize that they are bound to lose a trivially small bet, say a dollar, made on the outcome of some event, and compare this with the same individual having made a recklessly large bet, say five hundred dollars, on the same event. Focusing only on the anticipated monetary loss (and ignoring complexities such as possible issues of pride or embarrassment relating to the making, or failure to make, a correct prediction, and so on), we can suppose that in the first case, the person really wouldn’t care much about the prospect of losing a dollar, whereas in the second case, the person would care a great deal about losing five hundred dollars. In other words, losing a trivial amount would have no personal significance for the person, but losing the large amount would have a great deal of significance. From this, we can surmise that unlike the prospect of the large loss, which we would expect to result in some degree of an emotion (e.g., anxiety), the prospect of the trivial loss would evince no emotion at all (notwithstanding Ludwig van Beethoven’s Opus 129!). In general, it seems plausible to suppose that the magnitude of the personal significance of a construal is highly determinative of the intensity of any associated emotion.

The example above brings to light an important issue: as the size of the bet increases from a negligible amount towards a recklessly large amount there must come a point at which the prospect of losing the wager acquires some degree of personal significance for the individual (who, recall, is a person of modest means). In our OCC model we addressed this issue by proposing the idea of an emotion threshold—the point at which (for a particular individual, at a particular time, with respect to a particular emotion type) the magnitude of what we called the *emotion potential* of a cognition would be sufficient to allow an emotion to emerge [11] (pp. 215–281). This transition we viewed as a phase transition wherein at a certain point a *quantitative* change leads to a *qualitative* change as when, for example, a change in the temperature of H_2_O results in its transition from a liquid, qualitative, state (water) to a different, solid, qualitative state (ice). We proposed that when the quantitative dimension of emotion potential crosses the emotion threshold there is a transition from the qualitative state of an affect-free cognition to a different, affect-laden, qualitative state (an emotion). Furthermore, we realized that whereas conceiving of the quantitative dimension of the content of a cognition as emotion potential makes sense, conceiving of the quantitative dimension of the content of an emotion does not (because the potential has been realized). Accordingly, we proposed that when applied to the post-transition state, the quantitative dimension should be viewed as the intensity of the emotion. But, this surely is not right! A quantitative dimension cannot change as a function of whether a transition point has been reached—temperature remains temperature, regardless of the state that H_2_O is in. For this reason alone, in the case of emotions, the quantitative dimension is better conceived of as something like personal significance, which is just as applicable to the content of an emotion as to the content of the cognition that underlies it, while also not implying any pre- to post-transition identity change of the dimension itself. Reconceptualizing the quantitative dimension as personal significance has another important consequence: it allows for the fact that the intensity of an emotion is almost never determined by only the personal significance of its content. A discussion of the variables that determine the intensity of an emotion and how they differ as a function of emotion type is beyond the scope of this article, however it is noteworthy that an analysis of what it is for something to be an emotion can collide with issues relating to the intensity of emotions, a topic which although sometimes addressed [11,25,26] tends, curiously, to be largely ignored by emotion theorists.

### 2.3. Valence

In line with the idea that it is not possible to have an emotion about something while maintaining an attitude of indifference with respect to it is the fact that emotions are hedonic states. They are particular ways of feeling good or bad, necessarily positive or negative, but never neither [27]. Being valenced is a characteristic that differentiates emotions from another large and important class of mental states, namely, wants. Although in the emotion literature wants are sometimes discussed under the rubric of desires [28,29], most cognitively-oriented emotion theories tend to restrict their focus to goals, so that wants such as aspirations, wishes, hopes and the like are usually ignored. As Oatley and Jenkins put it [30] “a central tenet of most cognitive emotional theories is that emotions are elicited … by events *in relation to important goals*” (p. 65, italics added), a position that was quite explicit in the highly influential work of Richard Lazarus [31] who asserted that “there is no emotion without a goal at stake” (p. 51). Furthermore, specific proposals for checks on the congruence or incongruence of outcomes with one’s goals are a basic element of many appraisal theories (e.g., [32,33]). This limited focus on goals warrants a brief digression because although it is true that many emotions do indeed depend on the fate of goals, there are all kinds of wants that play an important role in emotions which have little or nothing to do with goals, meaning that a more nuanced analysis is needed. Goals are just a particular kind of want; they are future states over whose realization people can, at least in principle, have some material causal influence and that they therefore strive to attain by executing an action or series of actions—plans. Absent such conditions, one may want (or want not) something, but it cannot be a goal: having nice weather for your party cannot be a goal; nice weather is simply something that you want (hope for). Nor can you have the goal of it not raining, and it’s odd to say that you have the desire that it not rain. Again, raining is simply something that you want not to happen. We have many dormant wants in the form of principles, standards, and norms that we want upheld—we want people to tell the truth, and we want people not to suffer. An emotion like embarrassment need have nothing to do with goals. When sports fans report that they are embarrassed by their team’s performance, the problem is often less about their team losing and more about the quality of their team’s performance—the team ought to have made a better showing. Such judgments are prescriptive not descriptive and are justified by reference to standards—normative ideals, and moral or quasi-moral principles, not goals. Compared to goal-related wants, which are relatively impermanent and go away once realized, goal-independent wants are relatively stable; they are wants of a qualitatively different nature.

It might be tempting to think that wants must be valenced, but this temptation is diminished if one takes care to not confuse wants themselves with their objects and with their realization (or failure thereof). Suppose that you want the Green Party to win the election, or you want to take the subway to get to your place of work. The only locus of valence here lies in the object of the wants, not in the wants themselves. People generally want things that they deem to be positive and want-not things that they deem to be negative. Nor is there any question but that the realization or frustration of wants can give rise to emotions, but that is a different story. Wants themselves are neither positive nor negative. This even though wants, like emotions, have objects, and can vary in their strength, with such variations, incidentally, often contributing to the intensity of any emotions in which they play a role. And, to be sure, wants do play a major role in the intensity of a host of emotion types. The OCC model provides detailed specifications of some 20 emotion types that depend in one way or another on fulfilled or thwarted wants [11]. Some assuredly have to do with successes or failures with respect to (one’s own or others’) goals, but many have to do with approving (or disapproving) of the upholding (or not upholding) of standards and values that we want to be upheld and want not to be violated [11], all of which is testament to the importance of wants in giving rise to emotions, not to the valence of the wants themselves.

The requirement that emotions be valenced separates emotions from purely cognitive states, and in particular, it leads to the conclusion that surprise is not an emotion, a conclusion that many emotion theorists find troubling, not least because surprise is often assumed to be one of a small subset of alleged “basic emotions” that Keltner and colleagues dubbed the “basic six”—anger, fear, enjoyment, sadness, disgust, and surprise [1,34]. Whereas a detailed argument as to why surprise should not be considered to be an emotion is beyond the scope of this article, the gist of the argument is that pleasant surprises and unpleasant surprises are better conceived of as cases of surprise accompanied by positive or negative emotions [24] (pp. 53–57), and the fact that there are many cases of people being surprised by some fact but not caring one iota about that fact indicates that surprise cannot be intrinsically, necessarily, valenced (but see [35] for a dissenting opinion).

Finally, one might think that valence is essentially equivalent to personal significance in that the greater the absolute value of the valence the greater the personal significance. However, although the two are surely highly correlated, they are different variables as can be seen by considering the fact that there are many mental states that are not necessarily valenced but whose objects have high personal significance (e.g., beliefs, thoughts, wants, intentions).

### 2.4. Consciously Experienced

The next condition that I suggest is necessary for something to be an emotion some might consider contentious. It is the idea that, like pains and thoughts, emotions are a kind of conscious experience—they are states of which we are phenomenally aware—an idea that is a central tenet of the increasingly influential Constructionist theories of emotion [36]. This was also the view of Freud, who wrote: “It is surely of the essence of an emotion that we should feel it, i.e., that it should enter consciousness” [37] (p. 126). Along with Constructionists and Freud, I reject the possibility of unconscious emotions, just as I reject the notion of an “unfelt pain” proposed by some (e.g., [38]). I believe that some of the arguments in favor of unconscious emotions mistakenly equate behavioral concomitants of emotions with emotions themselves (e.g., [39]), while others fail to distinguish emotions from affective processing. For example, a famous study by Winkielman and Berridge [40] claiming to demonstrate the existence of unconscious emotions certainly provides convincing evidence of affective priming, but it provides no evidence that subjects actually experienced any specific emotions. As I have argued elsewhere [24] (pp. 52–53), such findings, interesting as they are, are more parsimoniously explained in terms of the effects of undifferentiated affect (which often is inaccessible to consciousness) rather than by postulating never evidenced discrete emotions. While affective processing is indisputably involved in the emergence of emotions and in the attachment of valence to previously unvalenced material, it is not the same thing as emotion.

Another way of saying that emotions are states of which we are necessarily aware is to say that an emotion is a (certain kind of) feeling. Admittedly, this raises the question of what feelings are, but whatever the answer to that question, I assume that it is true (indeed tautological) to say that feelings are necessarily felt, so one cannot have an unfelt feeling. From my perspective, with respect to the category of emotions, feelings are a superordinate category, so that all emotions are feelings, but not all feelings are emotions. For example, the feeling that we label “hunger” is not an emotion (it’s a drive—a kind of want), nor is the feeling of confusion, or the feeling of familiarity that characterizes recognition memory. I choose confusion and familiarity as examples of feelings not only because I do not consider them to be emotions, but because they have no obvious bodily components, making it difficult to conceive of them as forms of perception, which Peter Walla and colleagues believe to be important [21] (p. 9).

The requirement that we are necessarily aware of our emotions helps us distinguish emotions from wants (and want-nots) and beliefs. At any moment in time, we are completely unaware of the vast majority of our beliefs and of the things that we want. To be sure, some wants, notably drives such as hunger and thirst, are, it could be argued, necessarily in conscious awareness, but even if one accepts this, drives would fail to satisfy other requirements for being an emotion, such as the valence requirement and, arguably, intentionality, not to mention the fact that one could question whether they are even mental (as opposed to physical/bodily) states [23].

Finally, with respect to their being consciously experienced, we always have to be ready to address the question of what is going on when we say things like “I didn’t realize that I was angry until I sat down and thought about it” or “John is obviously jealous, but he just won’t admit it.” From my perspective, the answer here is quite simple. If I didn’t realize that I was angry until I sat down and thought about it, then I wasn’t angry until I sat down and thought about it! Some but not all of the elements of an anger emotion might have already been present—perhaps my feeling of anger had to await the right construal, which in turn might have depended on my thinking more deeply about the situation. Similarly for John’s alleged jealousy. Some but not all of the elements of jealousy might have been present—John maybe exhibited behaviors and was in a situation that would lead an observer to expect him to experience jealousy, but clearly, that hadn’t yet happened. John wasn’t (yet) jealous.

### 2.5. Insuppressible

In an important article [1] laying out key characteristics of his five “basic” emotions, Paul Ekman opined about what he called the “unbidden occurrence” of emotions, writing “one can not simply elect when to have which emotion” (p. 189). He then went on to point out that whereas it is quite normal to choose to put oneself in situations that one believes will evince certain emotions (e.g., deciding to go to a fun party), emotions themselves arise “unbidden.” I am in partial agreement with Ekman’s position, but I believe that there is a further constraint on the generality of what can be claimed in this regard because there is another way in which one can elect to have a particular emotion. At least to some extent, one can choose to have a cognition that one knows is likely to reinstate a past emotion or to generate a certain kind of emotion anew. For example, a person can choose to think again about the prestigious prize that they won in the past and consequently feel some pride again, or they can choose to recall how a colleague insulted them and thus become angry (again). In other words, intentionally recalling a past emotion-evoking situation can sometimes serve as a heuristic for generating a specific emotion, and sometimes merely imagining certain kinds of situations can have a similar effect. So, in general, the constraint on Ekman’s unbidden occurrence claim could be summed up by a generalization of what one might call a “How to be happy” principle—if you want to feel happy, think happy thoughts! In principle, this heuristic can be applied to negative emotions as well, although (except perhaps for method actors) people rarely choose to experience negative emotions on demand.

For reasons such as those just discussed, I prefer a cautious version of the unbidden occurrence characteristic, making the relatively weak claim that emotions are insuppressible in the sense that under normal conditions one cannot elect to spontaneously have any emotion one chooses. The intention of the normal conditions caveat is to exclude situations in which a person is somehow able to generate or reinstate an active cognition that is capable of subserving the chosen emotion. Furthermore, even if it is possible for people under such special conditions to sometimes choose to get themselves into a particular emotional state, other things being equal, it is not possible to get out of an emotional state by simply willing it to instantaneously terminate. This is not to deny that people can sometimes modulate the felt intensity and to some extent control the outward expression of (many of) their emotions, aspects of emotion control central to the area of emotion regulation [41]. My purpose here is not to elaborate on these issues but simply to make clear that in proposing that being insuppressible is a necessary characteristic of emotions my intention is to highlight the difficulty of exercising voluntary control over the initiation, termination, time course, and identity of emotions.

It is perhaps worth noting that from the perspective of appraisal theories, an emotion can end abruptly, even voluntarily, if the experiencer comes to believe that the underlying cognition is based on a misconstrual and therefore decides to abandon that cognition. Typically, this happens when one encounters new information as might happen, for example, when one learns that a person with whom one is angry for having neglected to fulfil a promise was in fact prevented from doing so because of being involved in a horrible accident, or when the young man whose pride was grounded in the belief that he had saved the child from drowning learns that in fact the child was never really in danger, thus leaving him nothing to be proud of. Importantly, realizing that the underlying cognition is based on a misconstrual is not sufficient. The person must also (be able to) abandon the cognition, something that sometimes one can choose to do, but not always. A person suffering from fear of flying may, as the plane prepares to take off, come to believe that nothing bad will happen, but is nevertheless incapable of abandoning the thought and thus remains fearful.

Being insuppressible is a characteristic that emotions share with moods and pains, but not with wants. For emotions, moods, and pains, unless one can interfere with the cause (abandoning the underlying cognition or taking a painkiller), the experience has to run its course, whereas for many wants one can choose to ignore them—people give up on aspirations all the time.

## 3. Two Inessential Characteristics of Emotions

At this point, a reader might be surprised that I have made no mention of two of the most frequently discussed aspects of emotions, namely, the role of bodily changes and the presence of facial expressions. But this is no accident. I believe that these two aspects of emotions are generally, but mistakenly, assumed to be necessary because of the biasing effects of frequency and salience—they occur frequently, and when they occur, because the emotions with which they are associated are usually quite intense, they are very noticeable. Consider first the idea that emotions always have a physiological component (variously referred to as emotions involving ANS activity, having a somatic component, or involving bodily changes or feelings thereof), an aspect of emotion orthodoxy for which we might have William James to thank. In 1884, James’ famous article “What is an emotion?” [42] appeared in the leading philosophy journal, *Mind*. It was to become one of the most influential articles in the history of emotion research, not because the core idea of the theory would become the standard theory (although for several decades after its publication, it was), but because the underlying assumption upon which the theory was based was taken as a given. The core idea proposed by James, and soon after by the Danish physician, Carl Lange (hence, the James-Lange Theory), was that an emotion is the feeling of bodily changes (the “bodily expression”) that results from “the perception of the exciting fact,” not, as common sense would have it, the other way around. The underlying assumption on which the core idea was based, and which, to this day, appears to be accepted without question by the majority of emotion theorists, is that (the feeling of?) bodily change is an essential component of emotions—that a distinguishing feature of emotions is that they necessarily have a somatic aspect.

What warrants this assumption? Although modern day scholars rarely point this out, early in James’ article, he wrote: “I should say first of all that the only emotions I propose expressly to consider here are those that have a distinct bodily expression.” He then went on, seemingly begrudgingly, to reveal some unease with his self-imposed restriction: “That there are feelings of pleasure and displeasure, of interest and excitement, bound up with mental operations, but having no obvious bodily expression for their consequence, would, *I suppose*, be held true by most readers” ([42] p. 189, italics added). Decades later, Walter Cannon, while acknowledging with obvious skepticism that the James-Lange theory was almost settled science, nevertheless, took it on, with a direct challenge to the core idea [43]. But also, as a final observation, he commented on the underlying assumption, noting that “James had to assume indefinite and hypothetical bodily reverberations in order to account for mild feelings of pleasure and satisfaction” ([43] p. 124).

By his own admission, James was only concerned with what he called the “standard” emotions—“strong” emotions which are accompanied by “a wave of bodily disturbance of some kind” [42] (p. 189). Never mind that his examples of such emotions (“surprise, curiosity, rapture, fear, anger, lust, greed, and the like”) included several states (surprise, curiosity, lust, greed) whose status as emotions one might reasonably question. What is important is that by constraining the theory to a category of states that have some particular property (strong emotions having “a wave of bodily disturbance”), the role of that property as constitutive to those states is guaranteed. It is, of course, often the case that a salient feature of strong emotions is that they are accompanied by bodily changes, but frequent and salient is not the same as necessary. It may well be that bodily changes are always present in members of a category that one might call “highly aroused states,” but that is not the same category as the category of emotions. It seems more reasonable to suppose that whereas some emotion experiences (e.g., fury over a vicious affront) can have a conspicuous bodily component, others (e.g., the quiet satisfaction of having finally solved a difficult puzzle) do not. Ergo, for something to be an emotion, it is not necessary that it involves some sort of felt bodily component, unless, of course, one excludes from the category of emotions, as James did, any states that are experienced without any (felt) bodily component.

The lesson to be learned here is that care has to be taken to avoid mistaking what is merely typically true or a very salient feature of emotions with what is necessary for something to be an emotion. It is my contention that this is exactly what happens when emotions are assumed to necessarily have a bodily component, and that Cannon was spot on when he raised concerns about the validity of this assumption in the case of mild emotions.

Much the same argument can be made with respect to facial expressions of emotions. For more than 50 years, the study of facial expressions, considered to be the most prototypical exemplars of “distinctive universal signals” [44], has been a central focus of human emotion research. Perhaps initially inspired by Darwin’s seminal book *The expression of the emotions in man and animals* [45], a focus on the “expressive” aspect of emotions as a means of communication to conspecifics has gained an inordinate amount of attention, with facial expressions often being taken to be a sine qua non of emotions. But again, while a recognizable facial expression is often a very conspicuous aspect of an emotion, frequent and salient is not the same as necessary. It seems to me that “no facial expression, no emotion” is just too radical! Furthermore, it is easy to demonstrate a double disassociation between facial expressions and emotions, and indeed between bodily changes and emotions for that matter. The fact that a facial expressions such as a grimace or a smile, or a bodily change such as an increase in heart rate or facial flushing can occur in the absence of emotions, and that emotions can occur without any such occurrences is clear indication that they are not necessary components of emotions.

## 4. Conclusions

I have proposed five characteristics which I believe are good candidates for necessary (and hopefully jointly sufficient) conditions for something to be an emotion. The determination of these characteristics has inevitably been a predominantly theoretical enterprise because we are trying to make more precise a concept whose use both in lay and scientific circles is extraordinarily vague—vague because the subjective nature of mental states in general and of emotions in particular precludes the possibility of identifying publicly observable membership criteria for the different categories that comprise them. The result of this enterprise is that for formal, scientific, purposes, we can consider an emotion to be an *insuppressible valenced mental state of which an individual is consciously aware and that is about something specific which is appraised as being of personal significance*.

At the outset of this article, I suggested that the goal of “defining” emotions should be to articulate a scientifically sound and clinically useful characterization of the emotion concept without doing serious violation to the vernacular use of the term *emotion*. I believe that to be useful, such a characterization should be succinct, and free of caveats and constraints on its generalizations. This means that it is unlikely that such a characterization can be achieved by taking bits and pieces from different proposals and integrating them into a unified representation that makes everybody happy. One such attempt was made some 45 years ago and resulted in the most prolix of definitions. After a review of close to 100 different accounts/definitions of emotions, many proposed and published by eminent scholars, P.R. Kleinginna and A.M. Kleinginna defined an emotion as “a complex set of interactions among subjective and objective factors, mediated by neural/hormonal systems, which can (a) give rise to affective experiences such as feelings of arousal, pleasure/displeasure; (b) generate cognitive processes such as emotionally relevant perceptual effects, appraisals, labeling processes; (c) activate widespread physiological adjustments to the arousing conditions; and (d) lead to behavior that is often, but not always, expressive, goal-directed, and adaptive” ( [46] p. 355). The contrast between my proposal and that of Kleinginna and Kleinginna couldn’t be more stark. Mine attempts to specify what must be the case for something to be an emotion. Theirs focuses on what can be or is often the case. Mine commits to emotions being valenced experiences, theirs does not. Mine considers as definitive only characteristics deemed to be necessary, eschewing those which are merely frequent or possible (e.g., behavioral consequences), theirs includes characteristics that are merely frequent or possible. In short, mine attempts to say what *is* (always) true of emotions, theirs says what *can* be or sometimes is true. Mine attempts to say what emotions are, theirs is about how emotions arise, what they generally do, and how they do it, finessing the prior and legitimate question of what it takes for something to be an emotion in the first place. But they are not unique in this respect. Few people stop to ask the question I have been asking about the criteria for membership of the category of emotions.

It is also interesting to compare my proposal with that of Peter Walla and colleagues in this Special Edition of this journal. They define an emotion as *an observable behavioral response that communicates an individual’s inner (feeling) state.* Admirable in its simplicity, this account views the presumed communicative function of emotions as a defining feature, while wanting to distinguish emotions from non-conscious processing (affection) and from subjective feeling, as well as from cognitive information processing. From my perspective, apart from my unwillingness to reject the idea that emotions are subjective experiences, both the idea that emotions are (necessarily) “observable” and that they are “behavioral responses” are very much at odds with our everyday conception of emotions. The requirement that emotions are observable implies that to claim that one cannot discern whether, on some particular occasion, an individual is angry or envious or disappointed and so on, is self-contradictory, which I think it is not. Meanwhile, the suggestion that emotions are behavioral responses obliterates the distinction between emotions and emotional expressions, rendering at least one of them superfluous. It is of course true that emotions often are communicated to others, but it is also true that they often are not, a fact which I believe is sufficient to establish the non-necessity of a communicative function. Furthermore, defining emotions in terms of their function of communicating inner feelings does not clearly distinguish emotions from language or from music, both of which really are observable behaviors that can be said to communicate an individual’s inner feelings (and other) states. As far as the role of emotions in transmitting information is concerned, it is perhaps worth noting that it has also been argued quite convincingly that emotions provide *internal* signals to an organism’s information processing mechanisms, redirecting attention and resources for the management of plans and goals in light of a constantly changing world [14,47].

Finally, the five characteristics I have proposed are of two kinds: those that must be present as an emotion emerges, and those that must be present after an emotion emerges. For an emotion to emerge there has to be an underlying cognition whose object has personal significance. Meanwhile, an emotion having emerged is always valenced, accessible to consciousness, and insuppressible. In the final analysis, if any one of these is not present, there is no emotion.

## Data Availability

No new data were created or analyzed in this study.
